# CNN and Attention-Based Joint Source Channel Coding for Semantic Communications in WSNs

**DOI:** 10.3390/s24030957

**Published:** 2024-02-01

**Authors:** Xinyue Liu, Zhen Huang, Yulu Zhang, Yunjian Jia, Wanli Wen

**Affiliations:** School of Microelectronics and Communication Engineering, Chongqing University, Chongqing 401331, China; 27168752@alu.cqu.edu.cn (X.L.); zhenhuang@cqu.edu.cn (Z.H.); yulu_zhang@stu.cqu.edu.cn (Y.Z.); yunjian@cqu.edu.cn (Y.J.)

**Keywords:** mobile edge computing, wireless sensor networks, semantic communications, attention mechanism, joint source channel coding, deep neural network

## Abstract

Wireless Sensor Networks (WSNs) have emerged as an efficient solution for numerous real-time applications, attributable to their compactness, cost-effectiveness, and ease of deployment. The rapid advancement of 5G technology and mobile edge computing (MEC) in recent years has catalyzed the transition towards large-scale deployment of WSN devices. However, the resulting data proliferation and the dynamics of communication environments introduce new challenges for WSN communication: (1) ensuring robust communication in adverse environments and (2) effectively alleviating bandwidth pressure from massive data transmission. In response to the aforementioned challenges, this paper proposes a semantic communication solution. Specifically, considering the limited computational and storage resources of WSN devices, we propose a flexible Attention-based Adaptive Coding (AAC) module. This module integrates window and channel attention mechanisms, dynamically adjusts semantic information in response to the current channel state, and facilitates adaptation of a single model across various Signal-to-Noise Ratio (SNR) environments. Furthermore, to validate the effectiveness of this approach, the paper introduces an end-to-end Joint Source Channel Coding (JSCC) scheme for image semantic communication, employing the AAC module. Experimental results demonstrate that the proposed scheme surpasses existing deep JSCC schemes across datasets of varying resolutions; furthermore, they validate the efficacy of the proposed AAC module, which is capable of dynamically adjusting critical information according to the current channel state. This enables the model to be trained over a range of SNRs and obtain better results.

## 1. Introduction

Wireless Sensor Networks (WSNs) [1,2] have emerged as highly effective solutions for a multitude of real-time applications owing to their compactness, cost-effectiveness, and ease of deployment. In recent years, the rapid development of 5G technology and mobile edge computing (MEC) [3,4] has facilitated the gradual transition of WSN devices towards large-scale deployment [5]. For instance, the deployment of numerous sensor nodes in urban areas enables real-time monitoring of environmental conditions, thereby offering crucial insights for urban planning and management. However, the resulting proliferation of data and the complexity of evolving communication environments introduce new challenges for WSN communications: (1) ensuring robust communication in poor Signal-to-Noise Ratio (SNR) conditions; (2) effectively alleviating bandwidth pressure amidst massive data transmission, particularly in high-density WSN scenarios.

Current WSN communication strategies employ traditional separated source channel coding methods, prioritizing accuracy and high fidelity at the physical layer. This often necessitates transmitting complete raw data, irrespective of content relevance, resulting in inefficiencies. Furthermore, this conventional approach is susceptible to the “cliff effect”, leading to communication failures in challenging conditions. This makes it difficult to fulfill high-precision task requirements in low SNR environments, a situation exacerbated in high-density WSNs. Deep Learning (DL)-based semantic communication offers a novel approach by employing Joint Source Channel Coding (JSCC) through Deep Neural Networks (DNNs) [6,7], focusing on transmitting semantic information rather than all data, thereby demonstrating potential in reducing data volume and enhancing communication reliability.

Recent preliminary research in the field of semantic communication has demonstrated its potential to effectively address the two aforementioned challenges in WSNs. Bourtsoulatze et al. [8] developed a JSCC scheme utilizing convolutional neural networks for wireless image semantic communication (ISC) that outperformed advanced separate source channel coding methods (JPEG+LDPC, JPEG2000+LDPC), particularly in low SNR environments. They also discovered that deep JSCC is immune to the “cliff effect”. To mitigate channel distortion from noise, certain approaches [6,7,9] have adopted generalized divisive normalization (GDN) and incorporated feedback mechanisms to enhance performance. However, these methods are limited to training at a fixed SNR and exhibit suboptimal performance in varying SNR conditions. Consequently, ref. [10] introduced a design featuring a single JSCC-encoder paired with multiple JSCC-decoders, where the decoder selection is contingent on the channel SNR. This design facilitates optimal model performance across a spectrum of SNRs, albeit at the expense of significant computational and storage demands, hindering its large-scale applicability in WSN devices. Therefore, the development of a single model adaptable to a wide range of SNRs is essential.

The advent of attentional mechanisms, particularly their successful application in visual tasks, offers a novel direction to tackle the aforementioned challenges. This approach [11] enhances feature learning in pivotal regions by emulating attention allocation processes observed in biological vision, concurrently suppressing the interference from non-essential information. Current research [12,13,14,15,16] indicates that while non-local attention adaptively adjusts feature representation and enhances model performance, it concurrently incurs considerable computational overhead. Conversely, the window attention mechanism [17] offers an efficient alternative, applying attention within a confined scope, thereby diminishing the model’s computational demands. Nevertheless, how to design a single model that can adapt to a wide range of SNR conditions remains an open research question, which will be explored in depth in this paper.

In this paper, we aim to address the problem of poor performance of a single model in semantic communication under different SNR conditions. Specifically, we propose an Attention-based Adaptive Coding (AAC) module for semantic communication and design a novel JSCC scheme based on this. The principal contributions of this paper are summarized as follows:

- We propose a flexible AAC module. Considering the resource-limited nature of WSN devices, it is able to capture the correlation between spatial neighboring elements to dynamically weight key local semantic information without sacrificing too many computational resources and is able to dynamically adjust the model output based on the current channel state information, which is capable of adapting/training a single model for a wide range of SNRs.
- We propose a novel JSCC model based on AAC modules and CNNs for ISC. Experimental results show that our model is more robust than the baseline model when compared to the current state-of-the-art methods, even in the case of channel mismatch.

**Figure 1:**
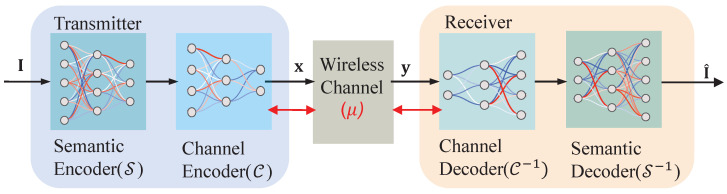
System model.

Figure 1 presents a detailed overview of an ISC system, comprising a transmitter and a receiver. The transmitter extracts semantic information from the input image using the semantic encoder. To guarantee the validity of the information, it is forwarded to the channel encoder prior to transmission. The encoded information is sent via wireless channels after power normalization, such as additive white Gaussian noise (AWGN) channels or fading channels to the receiver. Upon receiving the information, the receiver processes it sequentially through the channel decoder and the semantic decoder to reconstruct the image. In this paper, DNNs are employed to collaboratively design a semantic encoder/decoder and channel encoder/decoder for the ISC system. Furthermore, the wireless channel is conceptualized as a non-updatable layer, facilitating end-to-end optimization from the transmitter to the receiver.

## 2. System Model

Specifically, the input image of the transmitter is denoted as $I\in R^{H\times W\times C}$, where R signifies the set of real numbers, while *H*, *W*, and *C* represent the height, width, and color channels of the image, respectively. During the transmission stage, I is sequentially mapped by the semantic encoder S and the channel encoder C into an *L*-dimensional complex vector $x\in C^{L}$, where C denotes the set of complex numbers. In this context, S efficiently extracts semantic information from I, while C dynamically enhances this information in response to the current channel state (e.g., SNR), mitigating the adverse effects of the wireless channel. It should be noted that in this paper, we assume both communicating sides have the knowledge of the wireless channel’s SNR, denoted as μ. The bandwidth compression ratio, labeled as *R*, can be calculated as $R=\frac{L}{HWC}\in \left(0,1\right)$, where a smaller *R* indicates more compression. The above encoding process can be mathematically expressed as x=C(S(I),μ).

The wireless channel transmits the vector x, resulting in an output vector denoted as y, i.e.,

(1) $$\begin{array}{c} y=\left\{\begin{array}{cc} x+z, & \mathrm{for}\;\mathrm{AWGN}\;\mathrm{channel},\\ hx+z, & \mathrm{for}\;\mathrm{fading}\;\mathrm{channel}, \end{array}\right. \end{array}$$

where h∈C represents the channel gain, which is assumed to be a circularly symmetric complex Gaussian random variable with zero mean and unit variance, i.e., h∼CN(0,1), and $z∼\mathrm{CN}(0,\sigma_{n}^{2})$ is the complex Gaussian noise with zero mean and variance $\sigma_{n}^{2}$. Based on (1), μ can be calculated as

(2) $$\mu =\left\{\begin{array}{cc} 10\log_{10}\left(\frac{P}{\sigma_{n}^{2}}\right), & \mathrm{for}\;\mathrm{AWGN}\;\mathrm{channel},\\ 10\log_{10}\left(\frac{{P|h|}^{2}}{\sigma_{n}^{2}}\right), & \mathrm{for}\;\mathrm{fading}\;\mathrm{channel}, \end{array}\right.$$

where *P* is the transmit power of the transmitter. During the receiving stage, the receiver is equipped with a semantic decoder and a channel decoder, which are labeled as $S^{-1}$ and $C^{-1}$, respectively. Using the knowledge of μ, $S^{-1}$ and $C^{-1}$ decode y to a reconstructed image, denoted as $\hat{I}\in R^{H\times W\times C}$. Such a process can be mathematically expressed as $\hat{I}=S^{-1}\left(C^{-1}\left(y,\mu \right)\right)$.

Based on the above formulations, we define the overall DNNs as $N≜\{S,C,C^{-1},S^{-1}\}$. In this paper, we would like to train N to achieve a JSCC scheme for the ISC system. To evaluate the performance of the proposed JSCC scheme, we adopt the following two distortion metrics. The first is the PSNR metric [18] (in dB), defined as

(3) $$\begin{array}{c} \mathrm{PSNR}\left(I,\hat{I}\right)=\log_{10}\left(\frac{A^{2}}{||I-\hat{I}{\left|\right|}_{2}^{2}}\right), \end{array}$$

where *A* is the maximum possible value for a given pixel and ${∥\cdot ∥}_{2}$ is the $l_{2}$-norm operator. The other is the SSIM metric [19], defined as

(4) $$\begin{array}{c} \mathrm{SSIM}\left(I,\hat{I}\right)=\left(\frac{2\mu_{I}\mu_{\hat{I}}+v_{1}}{\mu_{I}^{2}+\mu_{\hat{I}}^{2}+v_{1}}\right)\left(\frac{2\sigma_{I}\sigma_{\hat{I}}+v_{2}}{\sigma_{I}^{2}+\sigma_{\hat{I}}^{2}+v_{2}}\right), \end{array}$$

where $v_{1}$ and $v_{2}$ are coefficients for numeric stability, and $\mu_{I}$ (resp. $\mu_{\hat{I}}$) and $\sigma_{I}^{2}$ (resp. $\sigma_{\hat{I}}^{2}$) are the mean and variance of I (resp. $\hat{I}$), respectively.

## 3. The Proposed JSCC Scheme

**Figure 2:**
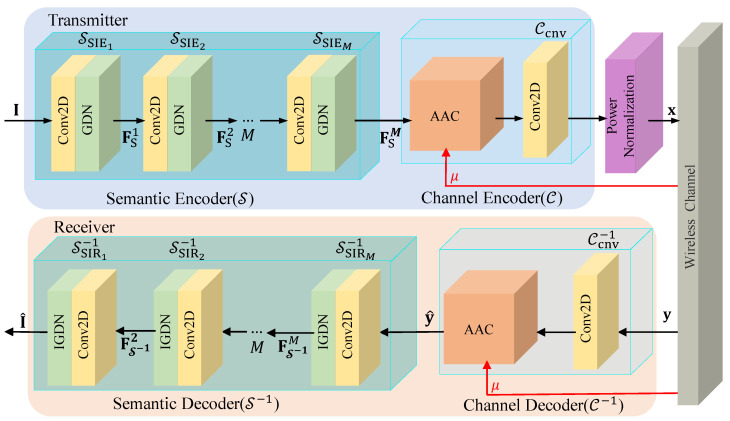
The neural network structure of the proposed JSCC scheme.

This section is dedicated to the development of the JSCC scheme for the ISC system. The comprehensive neural network architecture, represented by N, is illustrated in Figure 2. Initially, the semantic encoder neural network S, along with its corresponding decoder $S^{-1}$, is designed to extract and subsequently recover semantic information from the original image I. Subsequently, the channel encoder C and its decoder $C^{-1}$ are developed to mitigate the adverse effects typical of wireless channels. Benchmarking system performance necessitates the power normalization of encoded data before their transmission through the wireless channel. Ultimately, the comprehensive neural network N is realized through the integration of neural networks S, $S^{-1}$, C, and $C^{-1}$.

### 3.1. The Design of S and $S^{-1}$

For the effective extraction of image semantic information, Convolutional Neural Networks (CNNs) are utilized in the design of S and $S^{-1}$. As depicted in Figure 2, S comprises *M* Semantic Information Extraction (SIE) layers, denoted as $S_{\mathrm{SIE}}$. Each $S_{\mathrm{SIE}}$ layer encompasses a 2D convolutional layer, a Generalized Division Normalization (GDN) layer, and an activation function. The local perceptual capabilities of the convolutional layer enable the efficient extraction and recovery of semantic information such as colors, textures, shapes, and image contents. The nonlinear manipulation afforded by the GDN layer facilitates the extraction of more complex semantic information from the input image, ensuring spatially adaptive normalization. This aspect is pivotal in reducing spatial redundancy and more effectively capturing the image’s spatial structural information. The sequential layering of the SIE modules effectively extracts the image’s semantic information. The semantic information output by the semantic encoder, denoted as $F_{S}^{M}$, can be represented as

(5) $$F_{S}^{M}=S_{{\mathrm{SIE}}_{M}}\left(F_{S}^{M-1}\right),$$

when M=1, $F_{S}^{1}=S_{{\mathrm{SIE}}_{1}}\left(I\right)$. Analogous to S, $S^{-1}$ comprises *M* Semantic Information Reconstruction (SIR) layers, denoted as $S_{\mathrm{SIR}}^{-1}$. Each $S_{\mathrm{SIR}}^{-1}$ layer consists of a 2D deconvolution, an Inverse Generalized Division Normalization (IGDN) layer, and an activation function. $S^{-1}$ and $S_{\mathrm{SIR}}^{-1}$ represent the inverse processes of S and $S_{\mathrm{SIE}}$, respectively. In our design, the configuration of $S_{\mathrm{SIE}}$ and $S_{\mathrm{SIR}}^{-1}$ layers is symmetrical. The process of semantic decoding is formulated as

(6) $$F_{S^{-1}}^{m}=S_{{\mathrm{SIR}}_{m}}^{-1}\left(F_{S^{-1}}^{m+1}\right),m=1,2,\ldots ,M.$$

Here, $F_{S^{-1}}^{m}$ denotes the output from the *m*-th Semantic Information Reconstruction (SIR) layer within $S^{-1}$, with the output from the final SIR layer being the reconstructed image I. When m=M, the output $F_{S^{-1}}^{M}$ equates to $S_{{\mathrm{SIR}}_{M}}^{-1}\left(\hat{y}\right)$.

### 3.2. The Design of C and $C^{-1}$

**Figure 3:**
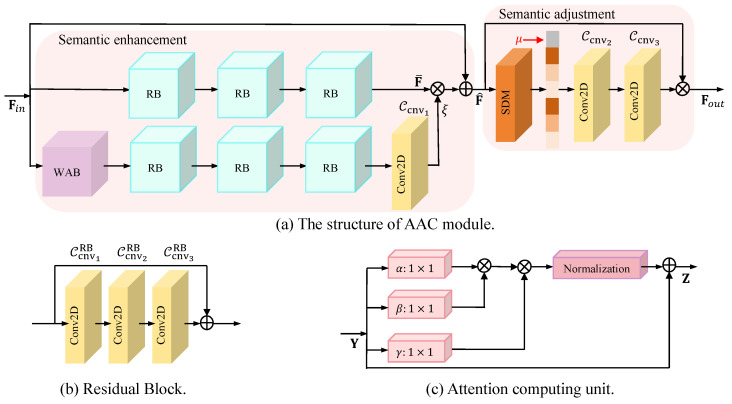
AAC module.

The channel encoder C comprises an AAC block and a 2D convolutional layer (labeled as $C_{\mathrm{cnv}}$). Similarly, the channel decoder $C^{-1}$ comprises an AAC block and a 2D deconvolution layer (labeled as $C_{\mathrm{cnv}}^{-1}$). Here, AAC is able to dynamically adjust the semantic information of the model output (resp. reconstruction) according to the current channel state to improve the quality of coding (resp. decoding), and $C_{\mathrm{cnv}}$ (resp. $C_{\mathrm{cnv}}^{-1}$) is used to adjust the transmitted (resp. received) information according to the bandwidth compression ratio. The AACs within C and $C^{-1}$ possess identical structures yet differ in their parameters. The architectural configuration of the AAC block is depicted in Figure 3a. This block comprises two components—semantic enhancement and semantic adjustment—both of which will be elucidated in detail.

Semantic enhancement: The semantic enhancement component consists of a Window Attention Block (WAB) and multiple Residual Blocks (RBs). In our architecture, the WAB is initially employed to empower the neural network to prioritize elements crucial to the current task, achieved by weighting key areas within the input feature map. This approach allows the neural network to focus its resources on processing specific regions of the image, like particular textures or edges, in more detail rather than treating the entire image uniformly. Such a mechanism enhances feature representation, particularly in scenes characterized by rich content or varied detail. Subsequently, the RBs assist the neural network in focusing on regions requiring improvement, as they are optimized for addressing reconstruction discrepancies during training. The detailed process is described below.

First of all, the input information of the AAC block, which is marked as $F_{in}$, passes through three RBs of the same structure, generating statistical information $\bar{F}$. As shown in Figure 3b, each RB consists of three convolutional layers (labeled as $C_{{\mathrm{cnv}}_{1}}^{\mathrm{RB}}$, $C_{{\mathrm{cnv}}_{2}}^{\mathrm{RB}}$, and $C_{{\mathrm{cnv}}_{3}}^{\mathrm{RB}}$), which help to improve the stability of GDN. The connection of multiple RBs can enhance the features of the input image to combat channel noise. At the same time, to generate the weight factor ξ, a WAB, three RBs, and a convolutional layer denoted as $C_{{\mathrm{cnv}}_{1}}$ are utilized. WAB is used to focus on high-contrast regions. Specifically, the feature map of the image is divided into windows of Q×Q in a non-overlapping way; then, it uses multi-head attention with a head number of *t* to compute the attention map in each window separately, where the attention computation unit is shown in Figure 3c. Suppose $Y_{i}^{q}$ and $Y_{j}^{q}$ are the pixels in the *i*-th row and *j*-th column of the *q*-th window. The *i*-th row output of the *q*-th window, which is labeled as $Z_{i}^{q}$, can be formulated as

(7) $$Z_{i}^{q}=\frac{W_{z}\sum_{\forall j}W_{\gamma }Y_{i}^{q}e^{W_{\alpha }W_{\beta }Y^{T}Y}}{\sum_{\forall j}e^{W_{\alpha }W_{\beta }Y^{T}Y}}+Y_{i}^{q},$$

where $W_{\alpha }$ and $W_{\beta }$ are cross-channel transforms, and $W_{\gamma }$ and $W_{z}$ are weight matrices. In order to improve the learning ability of the model, three RBs are used after WAB; then, a convolutional layer, followed by a Sigmoid function, is used, generating factor ξ∈[0,1]. Finally, by weighting and residual operations, $\hat{F}\in R^{H_{s}\times W_{s}\times C_{s}}$ is obtained, which has clearer semantic information. Here, $W_{s}$, $H_{s}$, and $C_{s}$ denote the semantically enhanced feature map width, height, and number of feature channels, respectively. The process can be expressed as $\hat{F}=F_{in}+\xi \bar{F}$.

Semantic adjustment: In order to effectively reduce the detrimental effects of the channel on the model and to improve the robustness (i.e., communication reliability) of the model in terms of the SNR over the range, we improve the channel attention mechanism to be able to dynamically adjust the more attended information based on the channel state information (i.e., SNR) of the changing wireless channel. Specifically, the sum of standard deviation and mean SDM(·) is first used to capture the global information within $\hat{F}$. Compared with the global average pooling operation, SDM(·) is able to better preserve information about relevant structures, textures, and edges, which are highly beneficial for enhancing image details (related to SSIM). For input vector $\hat{F}=\left[{\hat{F}}_{1},\ldots ,{\hat{F}}_{k},\ldots ,{\hat{F}}_{C_{s}}\right]$, the output of the *k*-th feature channel (defined as $z_{k}$) after SDM(·) can be expressed as

(8) $$\begin{array}{c} z_{k}=SDM\left({\hat{F}}_{k}\right)=\frac{1}{H_{s}W_{s}}\sum_{\lambda =1}^{H_{s}}\sum_{\nu =1}^{W_{s}}{\hat{F}}_{k}^{\lambda ,\nu }+\sqrt{\frac{1}{H_{s}W_{s}}\sum_{\lambda =1}^{H_{s}}\sum_{\nu =1}^{W_{s}}{\left({\hat{F}}_{k}^{\lambda ,\nu }-\frac{1}{H_{s}W_{s}}\sum_{\lambda =1}^{H_{s}}\sum_{\nu =1}^{W_{s}}{\hat{F}}_{k}^{\lambda ,\nu }\right)}^{2}}. \end{array}$$

After that, the obtained global information is connected with μ along the feature channel dimension, and two convolutional layers (denoted as $C_{{\mathrm{cnv}}_{2}}$ and $C_{{\mathrm{cnv}}_{3}}$) are used to predict the weighting factors; finally, the adjusted output is obtained by weighting $\hat{F}$, which can be expressed as

(9) $$\begin{array}{c} F_{out}=\hat{F}C_{{\mathrm{cnv}}_{3}}\left(C_{{\mathrm{cnv}}_{2}}\left(Concat\left(z_{1},\ldots ,z_{k},\ldots ,z_{C_{s}},\mu \right)\right)\right). \end{array}$$

Here, Concat(·) denotes the concatenation operation, $C_{{\mathrm{cnv}}_{2}}$ uses the ReLu activation function to learn the nonlinear relationship, and $C_{{\mathrm{cnv}}_{3}}$ uses the Sigmoid activation function to ensure that the weight factor is between 0 and 1.

### 3.3. The Training Algorithm

We employ mean square error (MSE) to measure the difference between the original image I and the reconstructed image $\hat{I}$. Therefore, the loss function is given by

(10) $$L=\frac{1}{N}\sum_{i=1}^{N}d\left(I,\hat{I}\right).$$

Here, *N* is the number of samples, $d\left(I,\hat{I}\right)=\frac{1}{n}{∥I-\hat{I}∥}^{2}$ is the mean squared-error distortion, and *n* represents the total number of pixels in the image. Despite the presence of noise and interference, the entire DNNs N can effectively learn and recover the transmitted information by minimizing the loss function (10).

Algorithm 1 describes the training process for the neural network N. The first step involves initializing the neural network parameters for N. Following this, the image I is input into the semantic encoder S, which produces its semantic information $F_{S}^{M}$. Subsequently, the channel encoder C and the power normalization operation transform $F_{S}^{M}$ into x, which will be transmitted through the wireless channel. Upon receiving the compressed information y from the wireless channel, the channel decoder $C^{-1}$ outputs the information $\hat{y}$. Based on this, the semantic decoding neural network $S^{-1}$ reconstructs the transmitted image $\hat{I}$. Finally, we employ the Adam optimizer [20] to update the parameters of N.
**Algorithm 1** Training Algorithm for N**Input:** 
The original image I and SNR μ.**Output:** 
The neural network N.1:Initialize the parameters in N.2:**Transmitter:**Perform semantic encoding process: S(I)→$F_{S}^{M}$.Perform channel encoding and power normalization process: $C(F_{S}^{M},\mu )$→x.3:Transmit x over the wireless channel to obtain y in (1).4:**Receiver:**Perform channel decoding process: $C^{-1}\left(y,\mu \right)$→$\hat{y}$.Perform semantic decoding process: $S^{-1}\left(\hat{y}\right)$→$\hat{I}$.5:Compute the loss function in (10).6:Train the neural network $\left\{S,C,C^{-1},S^{-1}\right\}$ using Adam optimizer.

## 4. Simulation Results

In this section, we will give the specific parameter settings. Following that, we will assess the performance of the proposed JSCC scheme, as well as representative baselines, through the examination of simulation results.

### 4.1. Simulation Settings

N consists of four SIE and four SIR blocks (i.e., M=4). Table 1 details the structural parameters of N—*input*, *output*, *k_size*, *stride*, and *Activation*, denoted as input dimensions, output dimensions, convolution kernel sizes, step sizes, and activation functions, respectively. As an additional note, the size of U is determined by the bandwidth compression ratio calculation [8], and the parameter settings are the same for all RBs in C and $C^{-1}$. The proposed JSCC scheme along with two benchmark JSCC schemes [9,21], designated as CA-Deep-JSCC, Deep-JSCC, and N-Deep-JSCC, respectively, were trained and tested across varying bandwidth compression ratios ($\frac{1}{6}$ and $\frac{1}{12}$), employing datasets of different resolutions (CIFAR-10 [22], ImageNet2012 [23], and Kodak [24]). PSNR and SSIM were used as evaluation criteria to react to the reconstructed image quality (i.e., communication reliability). All experiments were conducted on a PC with an Intel Core i7-10700 CPU@2.90 GHz and an NVIDIA RTX A4000 GPU.

### 4.2. Performance Evaluation

**Figure 4:**
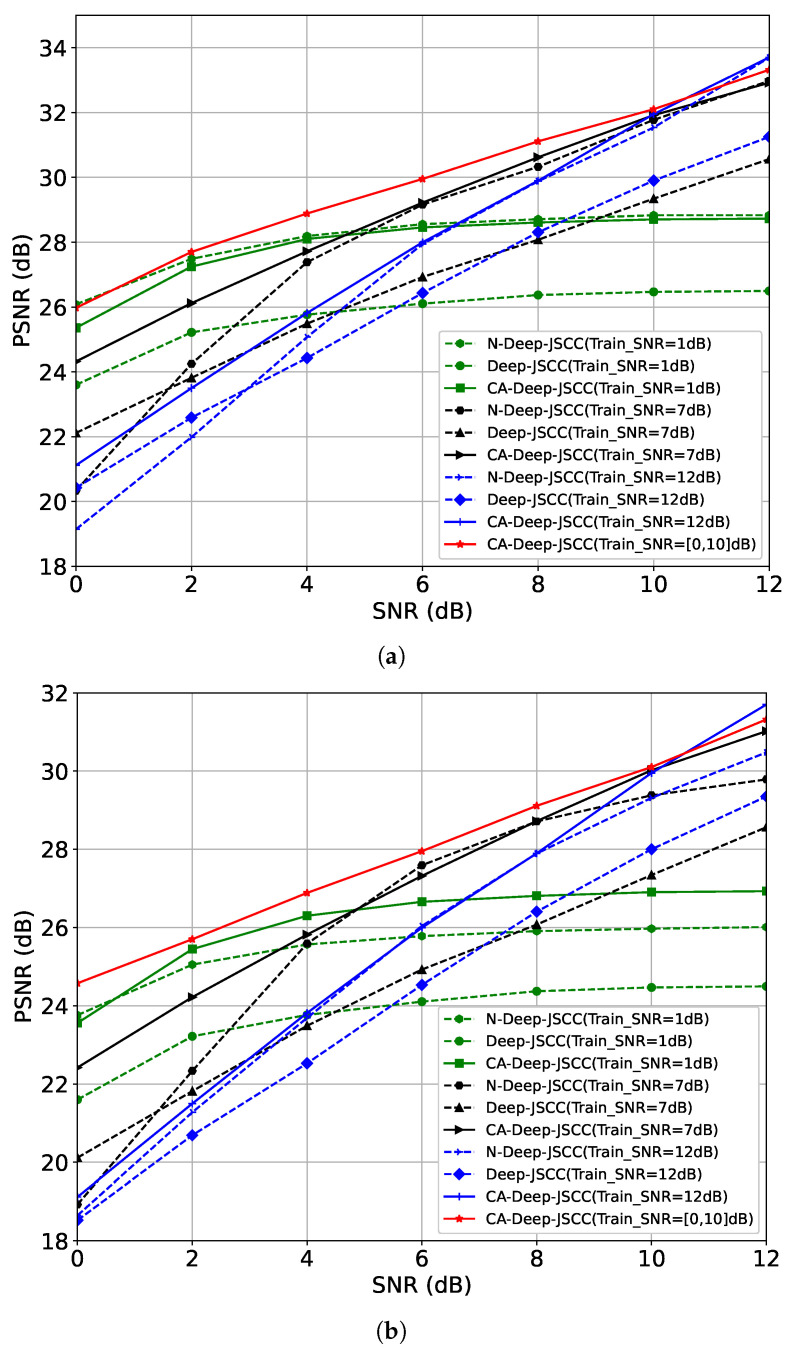
Model performance on CIFAR-10. Each curve of Deep-JSCC and N-Deep-JSCC was trained at a specific SNR. The curves of CA-Deep-JSCC were trained at 1 dB, 7 dB, and 12 dB with 0–10 intervals of 2 SNR. (**a**) Reconstruction distortion of Deep-JSCC, N-Deep-JSCC, and CA-Deep-JSCC on CIFAR-10, $R=\frac{1}{6}$. (**b**) Reconstruction distortion of Deep-JSCC, N-Deep-JSCC, and CA-Deep-JSCC on CIFAR-10, $R=\frac{1}{12}$.

**CIFAR-10 Performance Evaluation:** We use the CIFAR-10 dataset for training and testing, which contains 50,000 training images and 10,000 test images, and the image sizes are all 32×32. We set the batch_size to 128; the learning rate to $10^{-4}$; and use the Adam optimizer to train the models with SNRs of 1, 7, and 12, respectively, under bandwidth compression ratios of 1/6 and 1/12 for the model. Figure 4 shows the PSNR performance of CA-Deep-JSCC, Deep-JSCC, and N-Deep-JSCC for $R=\frac{1}{6}$ and $R=\frac{1}{12}$, where CA-Deep-JSCC is represented by the solid line and baselines by the dashed line. The red solid line shows the results of training on SNRs ranging from 0 to 10 intervals of 2. These results can be summarized as follows. Firstly, the PSNR of all schemes generally increases with the rising test SNR, attributable to the enhanced image reconstruction quality concurrent with signal quality improvement. Secondly, across all training instances with identical SNR and *R* values, the PSNR of the CA-Deep-JSCC scheme consistently exceeds that of the two baseline models, notably in the low SNR range. This finding indicates greater robustness of CA-Deep-JSCC under varied channel conditions. This is attributed to the fact that the proposed AAC module employs a window focusing mechanism, which enhances the semantic information that is focused on more. Furthermore, all schemes generally exhibit higher PSNR values at a compression ratio of $R=\frac{1}{6}$ compared to $R=\frac{1}{12}$, implying that higher compression ratios (or higher bandwidths) facilitate the transmission of more information through the channel, thereby improving image reconstruction quality. Finally, when trained within the SNR [0, 10] dB range, the proposed JSCC scheme demonstrates outstanding performance across all tested SNR levels. The superior performance is ascribed to the proposed AAC module’s capability to dynamically adjust semantic features in sync with real-time SNR, enabling CA-Deep-JSCC to be specifically trained within a certain SNR range, thereby markedly bolstering its robustness across different SNR scenarios.

**Figure 5:**
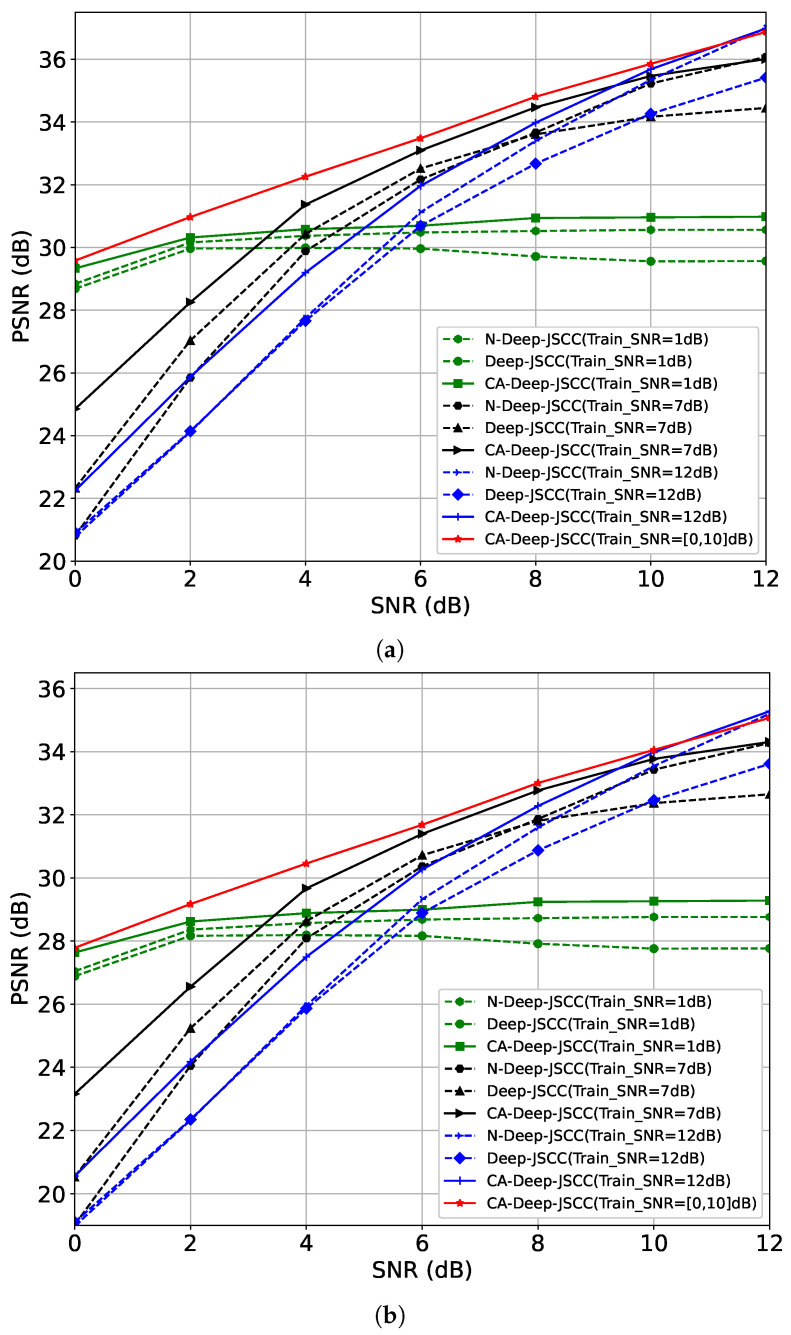
Model performance on Kodak. Each curve of Deep-JSCC and N-Deep-JSCC was trained at a specific SNR. The curves of CA-Deep-JSCC were trained at 1 dB, 7 dB, and 12 dB with 0–10 intervals of 2 SNR. (**a**) Reconstruction distortion of Deep-JSCC, N-Deep-JSCC, and CA-Deep-JSCC on Kodak, $R=\frac{1}{6}$. (**b**) Reconstruction distortion of Deep-JSCC, N-Deep-JSCC, and CA-Deep-JSCC on Kodak, $R=\frac{1}{12}$.

**Kodak Performance Evaluation:** To ensure the validity of higher resolution image data, we use the ImageNet2012 training set to train the model and evaluate it on Kodak, where the Kodak dataset contains 24 images of 512×768. The ImageNet2012 training set contains 1.3 million images of various resolutions; we filter the images whose size is larger than 128×128 (about 1.25 million images), which are then cropped to a patch of 128×128; set the batch_size to 128 and the learning rate to $10^{-4}$; and use the Adam optimizer for training. Similar to CIFAR-10, we train both models at $R=\frac{1}{6}$ and $R=\frac{1}{12}$, respectively, with fixed SNRs of 1, 7, and 12, and train CA-Deep-JSCC on a range of SNRs of 2 at intervals of 0 to 10. Figure 5 shows the reconstruction quality of both images on Kodak. First, the CA-Deep-JSCC scheme demonstrates a higher PSNR than the Deep-JSCC and N-Deep-JSCC schemes at nearly all SNR points under both bandwidth compression ratios, suggesting that the proposed scheme may be more effective in feature extraction and information transfer. Secondly, with a training SNR range of [0,10] dB, the proposed JSCC scheme exhibits the best performance at all test SNR points, which verifies the effectiveness of the AAC module on images of different resolutions. Finally, compared to the performance on the CIFAR-10 dataset, the model trained on the ImageNet2012 dataset demonstrates a higher PSNR on the Kodak dataset. This enhanced performance can be attributed to the following: (1) the diversity and complexity of the ImageNet2012 dataset, enabling the model to learn a broader feature representation; (2) the larger feature map size providing the model with more detailed information, which is important in ISC.

**Figure 6:**
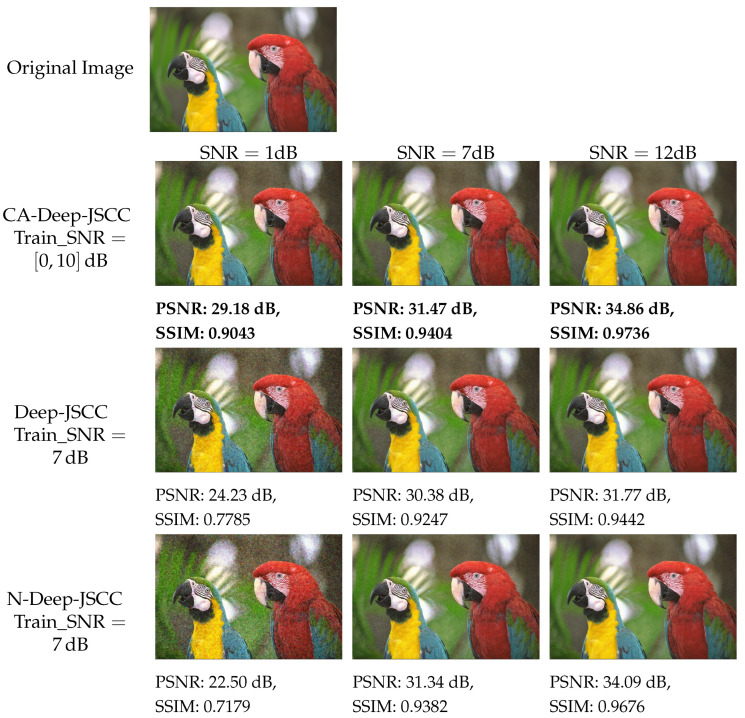
Visual comparison of three models (SNR = 1 dB, 7 dB, and 12 dB) for sample images of Kodak dataset under $R=\frac{1}{12}$.

To facilitate an intuitive visual comparison, a comparative analysis is presented between the CA-Deep-JSCC model, the Deep-JSCC model, and the N-Deep-JSCC model using sample images from the Kodak dataset in Figure 6. It can be observed that the quality of the reconstructed images of both the schemes improves as the SNR increases, which is quantified by the increase in PSNR and SSIM. The CA-Deep-JSCC scheme exhibits higher values of PSNR and SSIM in all SNR conditions, which indicates its superiority in the task of semantic communication of images.

## 5. Conclusions

This paper addresses the challenge of robustness in WSN communication through semantic communication solutions. We propose an AAC module for flexible integration into the ISC system, comprising a semantic enhancement component, utilizing the window attention mechanism, and a semantic adjustment component based on the channel attention mechanism. This module dynamically adjusts the focus on regions and semantic contents in response to the current channel state. Subsequently, in order to prove the effectiveness of AAC, a novel DNNs model, integrating CNN and AAC, is designed for ISC. Extensive simulations demonstrate that our proposed JSCC scheme surpasses existing state-of-the-art schemes in performance and can be trained across a range of SNRs, thereby enabling a single model to adapt to various SNR scenarios. This research advances the practical application of ISC schemes within the realm of WSN communication. Future research endeavors will focus on extending the JSCC scheme’s design to additional modalities—including text, audio, and video—and explore semantic-level data transfer mechanisms as well as model lightening techniques within WSNs with the objective of implementing a holistic semantic communication framework in WSNs.

## Figures and Tables

**Table 1:** The parameters of N.

Module	Layer		Input	Output	K_Size	Stride	Activation
S($S^{-1}$)	$S_{{\mathrm{SIE}}_{1}}$($S_{{\mathrm{SIR}}_{1}}^{-1}$)		3(256)	256(3)	9(9)	2(2)	PReLU(PReLU)
	$S_{{\mathrm{SIE}}_{2}}$($S_{{\mathrm{SIR}}_{2}}^{-1}$)		256(256)	256(256)	5(5)	2(2)	PReLU(PReLU)
	$S_{{\mathrm{SIE}}_{3}}$($S_{{\mathrm{SIR}}_{3}}^{-1}$)		256(256)	256(256)	5(5)	1(1)	PReLU(PReLU)
	$S_{{\mathrm{SIE}}_{4}}$($S_{{\mathrm{SIR}}_{4}}^{-1}$)		256(256)	256(256)	5(5)	1(1)	PReLU(PReLU)
C($C^{-1}$)	$C_{\mathrm{cnv}}$($C_{\mathrm{cnv}}^{-1}$)		256(U)	U(256)	3	1	PReLU(PReLU)
	AAC	$C_{{\mathrm{cnv}}_{1}}^{\mathrm{RB}}$	256	128	1	1	GELU
		$C_{{\mathrm{cnv}}_{2}}^{\mathrm{RB}}$	128	128	3	1	GELU
		$C_{{\mathrm{cnv}}_{3}}^{\mathrm{RB}}$	128	256	1	1	None
		$C_{{\mathrm{cnv}}_{1}}$	256	256	1	1	Sigmoid
		$C_{{\mathrm{cnv}}_{2}}$	257	256	1	1	ReLU
		$C_{{\mathrm{cnv}}_{3}}$	256	256	1	1	Sigmoid
		WAB	Q=4,t=8				

## Data Availability

CIFAR-10 dataset (https://www.cs.toronto.edu/~kriz/cifar.html, accessed on 12 July 2023); Kodak dataset (https://www.kaggle.com/datasets/sherylmehta/kodak-dataset, accessed on 16 October 2023); ImageNet2012 dataset (https://www.image-net.org/challenges/LSVRC/2012/, accessed on 16 October 2023).
